# Hepatitis B Virus PreS-Mutated Strains in People Living with HIV: Long-Term Hepatic Outcomes Following ART Initiation

**DOI:** 10.3390/v17081102

**Published:** 2025-08-11

**Authors:** Xianglong Lan, Yurou Wang, Min Liao, Linghua Li, Fengyu Hu

**Affiliations:** 1 Institution of Infectious Diseases, Guangzhou Eighth People’s Hospital, Guangzhou Medical University, Guangzhou 510440, China; lanxianglonglll@126.com (X.L.); wangyurou0628@126.com (Y.W.); liaomin_lm@163.com (M.L.); 2 Infectious Disease Center, Guangzhou Eighth People’s Hospital, Guangzhou Medical University, Guangzhou 510440, China; 3 Guangzhou Medical Research Institute of Infectious Diseases, Guangzhou Eighth People’s Hospital, Guangzhou Medical University, Guangzhou 510440, China; 4 Guangzhou Key Laboratory of Clinical Pathogen Research for Infectious Diseases, Guangzhou Eighth People’s Hospital, Guangzhou Medical University, Guangzhou 510440, China

**Keywords:** HIV, HBV, PreS deletion, point mutation, hepatic outcomes

## Abstract

In the modern era of HIV treatment, people co-infected with HIV and HBV still face poor liver outcomes, including liver fibrosis, liver cirrhosis, and hepatocellular carcinoma. We investigated baseline characteristics and long-term liver function outcomes in 435 people living with HIV and HBV co-infection, focusing on HCC-associated point mutations (PMs) and PreS region deletion mutations. PMs were present in 72.9% of participants and were associated with male predominance, lower HBV genotype C prevalence, reduced HBV DNA and HBeAg levels, and higher HBsAg and HBeAb positivity. However, PMs did not significantly impact liver function or fibrosis progression over six years of ART follow-up. In contrast, PreS deletions were found in 21.8% of cases and stratified into PreS1, PreS2, and PreS1+2 deletions. PreS2 and PreS1+2 deletions were linked to older age, higher HBsAg and AFP levels, elevated liver enzymes, and lower platelet counts. These groups also exhibited significantly worse liver fibrosis markers (APRI and FIB-4), with PreS2 deletions consistently showing the highest values throughout the follow-up. Despite the initial improvement with ART, patients with PreS2 and PreS1+2 deletions maintained higher fibrosis and cirrhosis risks over six years. In summary, while PMs were not predictive of liver disease progression, PreS deletion mutations (especially in the PreS2 region) were associated with poorer liver outcomes, indicating their potential as biomarkers for fibrosis risk in co-infected individuals with long-term ART.

## 1. Introduction

In HIV/HBV co-infection, although the implementation of antiretroviral therapy (ART) effectively suppresses HIV replication and concurrently inhibits HBV replication, the complete elimination of intrahepatic covalently closed circular DNA (cccDNA) remains challenging. As a result, residual HBV transcription and translation in hepatocytes may persist despite antiviral therapy [1,2], ultimately leading to enhanced hepatic inflammatory responses and accelerating the progression of liver disease [3,4]. Among individuals with HIV/HBV co-infection, liver-related mortality ranks second only to AIDS-related mortality following long-term ART, with 83% of liver-related deaths attributable to viral hepatitis [5].

Previous studies have reported that point mutations and deletion mutations within the HBV PreS region are associated with aggravated liver disease in HBV mono-infection, and have been identified as independent risk factors for hepatocellular carcinoma (HCC) [6,7,8,9,10,11]. Notably, patients harboring PreS deletions exhibit a higher incidence of end-stage liver disease [6,8]. Our research group has previously found that people co-infected with HIV and HBV harbor a high proportion of PreS deletion mutations within the viral quasispecies population [12], accompanied by delayed immune reconstitution and an increased incidence of Immunological Non-Responders (INRs) [13]. Based on the findings that HBV PreS mutations G2950A/G2951A/A2962G/C2964A significantly increased hepatocellular carcinoma risk and promoted hepatocarcinogenesis through endoplasmic reticulum stress and inflammatory signaling pathways [7], we selected these four point mutations for further investigation in our study. However, the long-term hepatic outcomes in people co-infected with HIV and HBV carrying a high proportion of PreS point mutations and deletions under sustained antiviral therapy remain inadequately characterized.

Therefore, the present study aims to evaluate the impact of PreS deletion mutations on long-term liver prognosis in people co-infected with HIV and HBV. This will be achieved through a comprehensive analysis of clinical data, laboratory parameters, and PreS region clonal sequencing.

## 2. Materials and Methods

### 2.1. Study Cohort

Among the HIV clinical cohort in Guangzhou Eighth People’s Hospital, 435 people living with HIV and HBV co-infection with initial anti-virus treatment between 2009 and 2019 were recruited, with excluded criteria at treatment baseline as follows: (1) HBV DNA < 1000 IU/L in plasma; (2) individuals with cancer or end-stage liver disease (ESLD); (3) individuals co-infected with other types of hepatitis virus (such as HAV, HCV, and HDV) and/or other apparent opportunistic infections; (4) individuals aged <18 years or >65 years, (5) pregnant or lactating women; (6) individuals with cardiovascular disease or renal failure; (7) no available plasma samples at baseline; and (8) unsuccessful PCR and/or sequencing of the HBV PreS region. Based on the presence of HCC-associated point mutations in the PreS region (G2950A, G2951A, A2962G, and C2964A), cases were classified into the point mutation group (PM) and the non-point mutation group (Non-PM). According to the location of deletion mutations, cases were further categorized as PreS1 region deletion (PreS1 del), PreS2 region deletion (PreS2 del), deletions in both PreS1 and PreS2 regions (PreS1+2 del), and without deletion in the PreS region (w/o del). This study’s protocol conformed to the Declaration of Helsinki and was approved by the Institutional Ethics Committee of Guangzhou Eighth People’s Hospital (Ethics Approval: 202033166). Written informed consent was obtained from individuals.

### 2.2. Clinical Data Collection and Serological Examination

Demographic, clinical characteristics, and laboratory data were collected from the clinical cohort.

### 2.3. Analysis of HBV PreS Region with T-A Cloning and Sequencing

Total DNA was extracted from a 200 μL serum sample collected from each patient using a fully automated nucleic acid extractor (Smart 32 Daan Genetics, Daan Gene Co., Guangzhou, China), with kit No. DA0623. The cloning method for the PreS region was the same as in our previous study [13]. PreS regions were aligned with the reference sequences (genotype C2, GenBank accession no. AB014378 and AB048705) using Bioedit (V.7.0) software. All mutations were checked manually. PreS1 region, nt 2848– 3204; PreS2 region, nt 3205–154. HCC-associated point mutations were G2950A, G2951A, A2962G, and C2964A

### 2.4. Statistical Analysis

Data analysis was conducted using SPSS version 25.0 (SPSS Inc., Chicago, IL, USA), with visualizations created in GraphPad Prism 9.5 (GraphPad Software, San Diego, CA, USA). Continuous data are presented as median values with interquartile ranges (IQRs). Categorical data are expressed as frequencies with percentages. Continuous variable comparisons employed Mann–Whitney U testing, while categorical variable analyses used chi-squared or Fisher’s exact tests. Two-tailed testing was applied throughout, with statistical significance defined as *p* < 0.05.

## 3. Result

### 3.1. Baseline Clinical Characteristics of People Living with HIV and HBV Co-Infection with HCC-Associated Point Mutations or Different Deletion Mutations in the PreS Region

A total of 435 people living with HIV and HBV co-infection were included in this study. The median age was 40 years (IQR: 33.5–50), with 376 participants (86.4%) being male. Regarding the HIV risk factor, 197 patients (45.3%) were infected through men who had sex with men (MSM), 198 (45.5%) through heterosexual transmission (HST), 7 (1.6%) through injection drug use (IDU), and 33 (7.6%) had unknown transmission routes (Table 1).

Among the 435 cases, 317 (72.9%) had HCC-associated point mutations (PMs) and 118 (27.1%) had no point mutations (Non-PMs). The PMs group exhibited significantly different characteristics compared to the Non-PM group: the male sex was more prevalent in the PM group (*p* = 0.027). HBV genotype C was significantly less frequent in the PM group (*p* < 0.001). HBV DNA levels were significantly lower in the PM group (median 7.62 vs. 8.06 Log10 IU/L, *p* = 0.005). HBsAg levels were significantly higher in the PM group (median 2815 vs. 1504 COI, *p* < 0.001). HBeAg levels were significantly lower in the PM group (median 72.37 vs. 1123.5 COI, *p* < 0.001), with HBeAg positivity also being significantly lower (*p* < 0.001). We conducted a stratified analysis based on HBeAg status and found that individuals with PMs consistently exhibited higher HBsAg levels, regardless of their HBeAg status. Notably, among HBeAg-positive individuals, those with PMs had significantly lower HBV DNA levels. However, in the HBeAg-negative group, the difference in HBV DNA levels between individuals with and without PMs was not statistically significant (Table S1). Conversely, HBeAb levels were significantly lower in the PM group (median 1.3 vs. 4.72 COI, *p* < 0.001), and HBeAb positivity was significantly higher in the PM group (*p* = 0.001). As for liver function indicators, no significant differences were observed. Baseline levels of HIV RNA, CD4, and CD8, and the CD4/CD8 ratio showed no statistically significant differences (Table 1).

PreS deletion mutations were identified in 95 patients (21.8%), with 46 patients (10.6%) having PreS1 deletions (PreS1 del), 22 (5.1%) having PreS2 deletions (PreS2 del), 27 (6.2%) having PreS1 and PreS2 deletions (PreS1+2 del), and 340 (78.2%) having no deletions (w/o del). Multiple deletion variants exist in the preS region, as reported in our previous study [12]. Here, we present five high-frequency deletion variants (Figure S1). Patients with different deletion patterns showed significant age differences (*p* = 0.014), with the PreS2 deletion group being older (median age: 50 years). HBV genotype C distribution varied significantly across deletion groups (*p* < 0.001). HBV DNA levels differed significantly between groups (*p* = 0.006), with the PreS2 deletion group showing the lowest levels (median 6.62 Log10 IU/L). HBsAg levels were significantly different across groups (*p* < 0.001), being the highest in the PreS1+2 deletion group (median 6915 COI). HBeAg levels and positivity rates were the lowest in the PreS2 deletion group, and varied significantly among deletion groups (*p* = 0.015 and *p* < 0.001). HBeAb levels and positivity rates also showed significant differences (*p* = 0.009 and *p* < 0.001). No significant differences were observed in HBsAb positivity or HBcAb positivity. Although baseline CD4 levels were lower in the PreS1 deletion group, there were no statistically significant differences in baseline HIV RNA, CD4, and CD8, or the CD4/CD8 ratio among the groups (Table 2).

Liver function parameters demonstrated significant associations with deletion mutations (Table 2). AFP levels were significantly elevated in patients with deletions (*p* < 0.001), PreS1+2 deletion group showing the highest levels (median: 7.19 μg/L). ALT and AST levels were significantly higher in deletion groups (*p* = 0.039 and *p* < 0.001), with the PreS1+2 del group showing the highest values. Total bilirubin was highest in the PreS2 deletion group (median: 12.18 μmol/L), and the difference was statistically significant (*p* = 0.020). Platelet counts were significantly lower in deletion groups (*p* = 0.014), particularly in PreS2 deletion patients (median: 124 × 10^9^/L). Liver fibrosis markers APRI and FIB-4 were significantly elevated in deletion groups (both *p* < 0.001), with PreS2 deletion patients showing the highest values (median: APRI 1.06; median: FIB-4 3.15).

### 3.2. HCC-Associated Point Mutations Had No Significant Impact on Liver Function Prognosis in People Living with HIV and HBV Co-Infection

**Figure 1:**
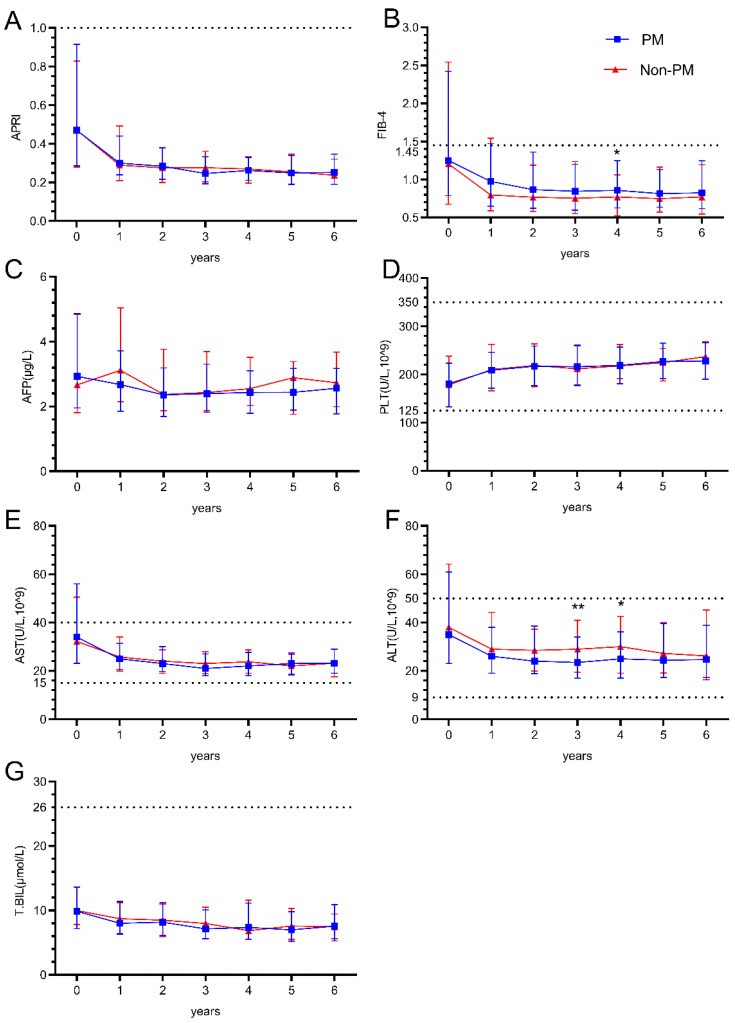
The impact of HBV PreS point mutations on hepatic outcomes in people living with HIV and HBV co-infection. (**A**,**B**) Longitudinal changes in liver fibrosis and cirrhosis risk scores (APRI/FIB-4) over six years after treatment; (**C**–**G**) longitudinal changes in liver function indicators (AFP, PLT, AST, ALT, and T.BIL) over six years after treatment; The dashed lines represent the reference range. Mann–Whitney U test for comparison between groups. *p* < 0.05 shows the difference is statistically significant. * *p* < 0.05, ** *p* < 0.01.

During the 6-year follow-up period after ART initiation (not all of the 435 patients were observed for the full 6-year period; the number of data points at each time point is shown in Supplementary Table S2), hepatic parameters showed distinct patterns between the PM and Non-PM groups. APRI values demonstrated a sharp decline from baseline to year 1 in both groups, then remained stable throughout the follow-up period with no significant differences between groups (Figure 1A). FIB-4 values showed a similar pattern of initial decline and stabilization, with a significant difference observed at year 4 (*p* = 0.042), where the Non-PM group maintained slightly lower values (Figure 1B).

ALT levels showed significant differences at years 3 and 4 (*p* = 0.009 and *p* = 0.036). Both groups demonstrated a decline from baseline values (median: 35 and 38 U/L) to lower levels (median: 26 and 29 U/L) by year 1, which were maintained throughout follow-up (Figure 1F). AST levels followed similar trajectories with an initial decline from baseline and stabilization at lower levels, without significant between-group differences (Figure 1E). Total bilirubin levels remained stable around 8–10 μmol/L throughout the follow-up period in both groups (Figure 1G). AFP levels showed slight fluctuations but remained within the range of 2.5–3.0 μg/L across all time points without significant differences (Figure 1C). Platelet counts demonstrated a gradual increase from baseline values (median: 181 and 178 × 10^9^/L) to higher levels (median: 228 and 237 × 10^9^/L) by year 6 in both groups (Figure 1D).

### 3.3. Deletion Mutations in the PreS2 Region Exacerbate the Risk of Liver Fibrosis and Cirrhosis in People Living with HIV and HBV Co-Infection

**Figure 2:**
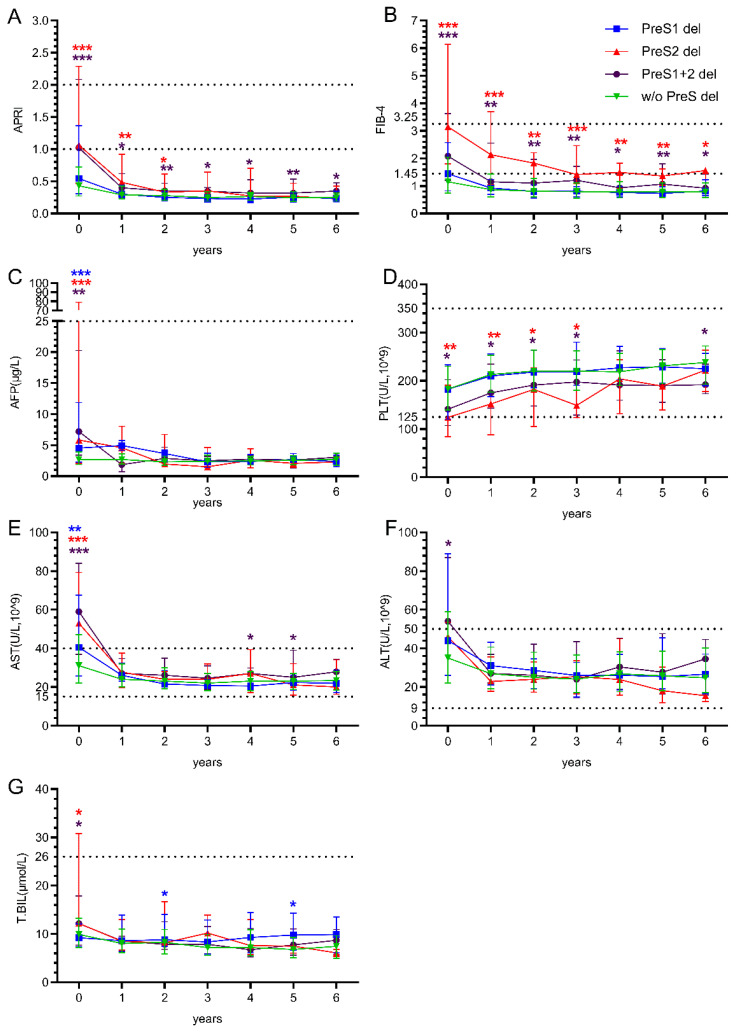
The impact of HBV PreS deletion mutations on hepatic outcomes in people living with HIV and HBV co-infection. (**A**,**B**) Longitudinal changes in liver fibrosis and cirrhosis risk scores (APRI/FIB-4) over six years after treatment; (**C**–**G**) longitudinal changes in liver function indicators (AFP, PLT, AST, ALT, and T.BIL) over six years after treatment; The dashed lines represent the reference range. Mann–Whitney U test for comparison between groups. *p* < 0.05 shows the difference is statistically significant. * *p* < 0.05, ** *p* < 0.01, and *** *p* < 0.001. Blue asterisks indicate the comparison between “PreS1 del” and “w/o del”; red asterisks indicate the comparison between “PreS2 del” and “w/o del”; purple asterisks indicate the comparison between “PreS1+2 del” and “w/o del”.

Patients with different PreS deletion patterns showed markedly distinct long-term outcomes. APRI values at baseline were significantly elevated in the PreS2 deletion group (median: 1.06, IQR 0.5–2.09) and PreS1+2 deletion group (median: 1.02, IQR 0.6–1.5) compared to the w/o deletion group (below the risk range). Significant differences persisted at years 1, 2, 3, 4, 5, and 6, with the PreS1+2 deletion group consistently showing the highest values throughout the follow-up (Figure 2A). FIB-4 values demonstrated the most pronounced differences, with the PreS2 and PreS1+2 deletion groups showing markedly elevated baseline values (median: 3.15, almost reached the high-risk range) compared to the w/o deletion group. Significant differences were maintained at years 1, 2, 3, 4, 5, and 6, with the PreS2 deletion group consistently exhibiting the highest values throughout the follow-up period (Figure 2B). Although AFP levels were significantly higher in the deletion groups at baseline, they remained consistently below 25 during the treatment period (Figure 2C). Platelet counts showed initial recovery patterns across all groups, with values increasing from baseline to the reference value range by year 6, but PreS2 and PreS1+2 deletion groups remained significantly lower at baseline and during years 1 to 3 of treatment without significant between-group differences in years 4–6 (Figure 2D).

AST levels demonstrated significant baseline differences among deletion groups, with PreS2 deletion patients showing the highest values. All groups showed an improvement over time, with convergence toward normal ranges by years 1–6 (Figure 2E). ALT levels showed less pronounced differences among deletion groups during the follow-up, with all groups maintaining relatively stable values after the initial year (Figure 2F). Total bilirubin levels remained stable across all deletion groups throughout the 6-year follow-up period. A significant increase was observed in the PreS1 deletion group in the second and fifth years of treatment (Figure 2G).

## 4. Discussion

Our study included 435 individuals co-infected with HIV and HBV and found that the prevalence of HCC-associated point mutations was 72.9%. These point mutations were predominantly of the HBV genotype B (84.5%), which differs from previous findings in HBV mono-infected individuals, where genotype C–related mutations were more common [7]. We found that the point mutations reduced HBV DNA and HBeAg levels and showed higher HBsAg and HBeAb positivity. However, some studies have indicated that point mutations do not result in significant changes in HBV DNA or HBsAg levels [14]. In fact, certain studies have reported that T123A/C/K/S and P142L/R/S/T mutations in the PreS region lead to decreased HBsAg secretion due to intracellular accumulation [15]. The two studies focused on different mutation sites and reported inconsistent observations; however, both found that point mutations affected HBV DNA and HBsAg.

The prevalence of PreS region deletion mutations was 21.83%. Although genotype C was less prevalent among the co-infected individuals (36.6%), the majority of those with PreS deletions were infected with genotype C (67.4%). A 2017 study from Guangxi [16] similarly reported a PreS deletion mutation rate of 23% among 61 HIV/HBV co-infected individuals, with genotype C being more common than genotype B. Given that HBV genotypes B and C may differ in pathogenic potential, and that genotype C infection is associated with higher incidences of chronic hepatitis, liver cirrhosis, and hepatocellular carcinoma compared to genotype B [17], these findings may also be influenced by the interactions between HIV and HBV.

We observed distinct patterns in immune parameter restoration across different mutation types over the 6-year follow-up period. For point mutations (PM vs. Non-PM), both groups showed comparable immune reconstitution trajectories, remaining relatively stable throughout the observation period (Figure S2A–C). In contrast, deletion mutations exhibited more heterogeneous recovery patterns (Figure S2D–F). These findings suggest that while HCC-related point mutations do not substantially impact long-term immune reconstitution, deletion mutations are associated with differential immune recovery patterns, as discussed in detail in our previous studies [13]. Recent studies have established that HBV PreS deletion is positively associated with liver fibrosis progression in chronic HBV-infected patients, with preS2 deletions serving as warning indicators for liver fibrosis progression [8,18,19]. Our baseline data showing significantly elevated APRI and FIB-4 scores in patients with PreS deletions, particularly PreS2 deletions (APRI: 1.06, FIB-4: 3.15), strongly support this association. The age distribution showing older patients in the PreS2 deletion group (median age: 50 years) is consistent with the progressive nature of fibrosis development over time.

Longitudinal studies on young HIV patients have demonstrated the slow progression of APRI and Fib-4 scores over time, with liver fibrosis scores remaining elevated in HIV-HBV patients regardless of HBsAg status [20]. Our follow-up data showing persistently elevated fibrosis markers in deletion groups, with statistical significance maintained through 6 years of follow-up, reinforce the prognostic importance of these mutations.

The frequency of PreS2 deletions has been reported to be higher in HBV coinfected patients with genotypes A and C, though not always reaching statistical significance [21]. Our finding of significantly higher HBV genotype C prevalence in deletion groups (76% overall in deletion patients vs. 28% without deletions) provides robust evidence for this genotype-specific mutation pattern.

HBsAg persistence has been correlated with mutations and deletions in envelope regions that play key roles in immune recognition, suggesting that envelope variability could favor immune escape [22]. The elevated HBsAg levels observed in PreS deletion groups, particularly PreS2 and PreS1+2 deletions, support this mechanism of immune evasion.

The limitation of this study is that prognosis was assessed solely through non-invasive clinical examinations, without the direct observation of liver pathology. Additionally, the follow-up endpoint did not include progression to hepatocellular carcinoma, which will be the focus of our future research.

In conclusion, the longitudinal nature of this study, extending to 6 years of follow-up, addresses a critical gap in understanding the long-term clinical consequences of HBV mutations in HIV coinfection. The persistent elevation of fibrosis markers and liver enzymes in mutation groups suggests that these viral variants may require more intensive monitoring and potentially modified treatment approaches.

## Figures and Tables

**Table 1:** Basic clinical information for people living with HIV and HBV co-infection with point mutations and without point mutations.

	Overall ^a^	PM	Non-PM	*p* Value
	(*n* = 435)	(*n* = 317)	(*n* = 118)	
AGE	40(33.5–50)	41(34–50)	38(33.25–48.75)	0.184
Sex (male, %)	376(86.44)	267(84.23)	109(92.37)	**0.027**
HIV risk factor (*n*, %)				
MSM	197(45.29)	138(43.53)	59(50)	0.229
HST	198(45.52)	152(47.95)	46(38.98)	
IDU	7(1.61)	6(1.89)	1(0.85)	
NA	33(7.59)	21(6.62)	12(10.17)	
HIV				
HIV RNA (Log10, copies/mL)	5.47(5.08–5.92)	5.47(5.09–5.92)	5.49(5.03–5.86)	0.567
CD4 (cells/μL)	172(45–302)	169(45–289)	190(47.25–336.75)	0.279
CD8 (cells/μL)	739(506.5–1095)	728(505–1082)	874.5(537.75–1148.75)	0.246
CD4/CD8	0.19(0.08–0.33)	0.19(0.08–0.33)	0.2(0.08–0.35)	0.694
HBV				
HBV genotype (C, %) ^b^	159(36.55)	49(15.46)	110(93.22)	**<0.001**
HBV DNA (Log10, IU/L)	7.74(6.68–8.63)	7.62(6.6–8.53)	8.06(7.25–8.7)	**0.005**
HBsAg (COI)	2426(1220.5–6604.5)	2815(1399–6984)	1504(763.28–4735.25)	**<0.001**
HBsAb (+, %)	7(1.61)	5(1.58)	2(1.69)	0.931
HBeAg (COI)	263.2(0.09–1395.5)	72.37(0.09–1348)	1123.5(11.24–1441.5)	**<0.001**
HBeAg (+, %)	276(63.45)	183(57.73)	93(78.81)	**<0.001**
HBeAb (COI)	1.78(0.02–5.98)	1.3(0.01–5.71)	4.72(0.85–6.55)	**<0.001**
HBeAb (+, %)	176(40.46)	143(45.11)	33(27.97)	**0.001**
HBcAb (+, %)	420(96.55)	308(97.16)	112(94.92)	0.254
Hepatic markers				
AFP (μg/L)	2.88(1.94–4.82)	2.93(1.97–4.82)	2.67(1.81–4.8)	0.224
ALT (U/L)	36(23–61)	35(23–61)	38(23–63.25)	0.755
AST (U/L)	33(23.1–55.1)	34(23.2–56)	32.2(23.25–49.75)	0.500
T.BIL (μmol/L)	9.96(7.33–13.59)	9.87(7.19–13.57)	9.99(7.82–13.58)	0.564
PLT (10^9^/L)	181(133–228)	181(133–224)	178.5(132.75–236.75)	0.694
APRI	0.47(0.28–0.89)	0.47(0.29–0.91)	0.47(0.28–0.83)	0.577
FIB-4	1.24(0.76–2.4)	1.25(0.79–2.42)	1.2(0.68–2.52)	0.264

Data are presented as case numbers (percentage, %) or median (P_25_–P_75_). *P* values are determined *using* the *chi*-*squared test or Fisher’s exact test* for categorical variables and the *Mann–Whitney U test* for continuous variables. A *p* value of less than 0.05 represents a statistically significant difference. ^a^: The present study utilized the same cohort as the previous investigation (OFAF377); ^b^: only HBV genotypes B and C were ultimately included in this study. Abbreviations: PM: with point mutations; Non-PM: non-point mutation group; MSM: men who have sex with men; HST: heterosexual; IDU: injection drug user; NA: not available; COI: cut-off index.

**Table 2:** Basic clinical information for people living with HIV and HBV co-infection between different PreS region deletions.

	Overall ^a^	PreS1 del	PreS2 del	PreS1+2 del	w/o del	*p* Value
	(*n* = 435)	(*n* = 46)	(*n* = 22)	(*n* = 27)	(*n* = 340)	
AGE	40(33.5–50)	40(35–47.75)	50(42.25–56)	43(36–55.5)	39(33–49)	**0.014**
Sex (male, %)	376(86.44)	9(19.57)	3(13.64)	6(22.22)	41(12.06)	0.284
HIV risk factor (*n*, %)						
MSM	197(45.29)	19(41.3)	5(22.73)	10(37.04)	163(47.94)	0.136
HST	198(45.52)	21(45.65)	13(59.09)	13(48.15)	151(44.41)	
IDU	7(1.61)	2(4.35)	0(0)	0(0)	5(1.47)	
NA	33(7.59)	4(8.7)	4(18.18)	4(14.81)	21(6.18)	
HIV						
HIV RNA (Log10, copies/mL)	5.47 (5.08–5.92)	5.46 (4.91–5.93)	5.54 (4.94–6.17)	5.6 (5.09–5.82)	5.46 (5.11–5.92)	0.934
CD4 (cells/μL)	172 (45–302)	106 (14.25–282)	159 (55.25–231.75)	167 (45–260)	184.5 (52–311.25)	0.064
CD8 (cells/μL)	739 (506.5–1095)	697 (492.75–1007.5)	681 (360.25–830.75)	697 (522.5–1058)	784 (522.25–1120.5)	0.176
CD4/CD8	0.19 (0.08–0.33)	0.14 (0.04–0.31)	0.21 (0.11–0.35)	0.15 (0.07–0.27)	0.2 (0.08–0.34)	0.193
HBV						
HBV genotype (C, %) ^b^	159(36.55)	35(76.09)	16(72.73)	13(48.15)	95(27.94)	**<0.001**
HBV DNA (Log10, IU/L)	7.74 (6.68–8.63)	7.93 (7.13–8.7)	6.62 (5.55–7.59)	7.55 (6.55–8.61)	7.76 (6.8–8.7)	**0.006**
HBsAg (COI)	2426 (1220.5–6604.5)	2843.5 (1165–6329.75)	5984.5 (2745–6576)	6915 (4286.5–7765)	2079 (1137.5–6271.5)	**<0.001**
Deletion mutation (frequency, %)	-	41.11 (21.39–66.78)	100 (95.34–100)	84.21 (44.61–100)	-	-
HBsAb (+, %)	7(1.61)	1(2.17)	0(0)	2(7.41)	5(1.47)	0.149
HBeAg (COI)	263.2 (0.09–1395.5)	708.3 (49.99–1295.25)	2.11 (0.09–61.11)	59.21 (3.89–712.5)	456.4 (0.09–1435.5)	**0.015**
HBeAg (+, %)	276(63.45)	42(91.3)	12(54.55)	21(77.78)	201(59.12)	**<0.001**
HBeAb (COI)	1.78 (0.02–5.98)	3.04 (1.26–5.63)	0.21 (0–1.5)	1.24 (0.63–2.69)	2.65 (0.01–6.21)	**0.009**
HBeAb (+, %)	176(40.46)	9(19.57)	22(100)	27(100)	144(42.35)	**<0.001**
HBcAb (+, %)	420(96.55)	45(97.83)	22(100)	27(100)	326(95.88)	0.488
Hepatic markers						
AFP (μg/L)	2.88 (1.94–4.82)	4.52 (2.39–11.15)	5.81 (2.13–71.85)	7.19 (3.53–16.6)	2.69 (1.91–3.87)	**<0.001**
ALT (U/L)	36 (23–61)	44 (26.5–86.75)	46 (26.75–52)	54 (30–82.5)	35 (22–59)	**0.039**
AST (U/L)	33 (23.1–55.1)	40.5 (26–65.5)	53 (40.25–77.5)	59 (38.5–82)	31 (22–47)	**<0.001**
T.BIL (μmol/L)	9.96 (7.33–13.59)	9.22 (7.38–11.31)	12.18 (8.78–28.02)	12.15 (7.83–17.6)	9.87 (7.21–13.24)	**0.020**
PLT (10^9^/L)	181 (133–228)	182.5 (122.75–229.5)	124 (91.25–184.75)	141 (108–199)	183 (139–230)	**0.014**
APRI	0.47 (0.28–0.89)	0.54 (0.32–1.27)	1.06 (0.5–2.09)	1.02 (0.6–1.5)	0.43 (0.27–0.72)	**<0.001**
FIB-4	1.24 (0.76–2.4)	1.44 (0.83–2.56)	3.15 (2.08–5.65)	2.1 (1.2–2.73)	1.15 (0.74–2.06)	**<0.001**

Data are presented as case numbers (percentage, %) or median (P_25_–P_75_). *P* values are determined *using* the *chi*-*squared test or Fisher’s exact test* for categorical variables and the *Kruskal–Wallis H test* for continuous variables of groups. Frequency and percentage of the deletion mutations means the proportion of clones harboring deletion mutations relative to the total clone population. A *p* value of less than 0.05 represents a statistically significant difference. ^a^: The present study utilized the same cohort as the previous investigation (OFAF377); ^b^: only HBV genotypes B and C were ultimately included in this study. Abbreviations: PreS1 del: with PreS1 deletion; PreS2 del: with PreS2 deletion; PreS1+2 del: with PreS1 and PreS2 deletions; w/o del: without PreS deletion; MSM: men who have sex with men; HST: heterosexual; IDU: injection drug user; NA: not available; COI: cut-off index.

## Data Availability

The raw data supporting the conclusions of this article will be made available by the authors on request.

## Supplementary Materials

The following supporting information can be downloaded at: https://www.mdpi.com/article/10.3390/v17081102/s1, Table S1: The Relationship Between Point Mutations and HBV DNA and HBsAg Levels in HIV/HBV Co-infected Patients with Different HBeAg Statuses; Table S2: Number of Available Data for Each Indicator at Each Follow-up Time Point; Figure S1: Diagram of Five Common High-Frequency Deletion Mutant Strains; Figure S2: The impact of HBV Point Mutations and PreS deletion mutants on immune reconstitution in People living with HIV and HBV co-infection.
