# Supplementary material for: Hepatitis B Virus PreS-Mutated Strains in People Living with HIV: Long-Term Hepatic Outcomes Following ART Initiation
Source: Viruses. 2025 Aug 11;17(8):1102. doi: 10.3390/v17081102 (PMC12390677; doi:10.3390/v17081102)
Supplement: Supplementary file 1 [file viruses-17-01102-s001.zip › viruses-3759719-supplementary.pdf]

Table S1. The Relationship Between Point Mutations and HBV DNA and HBsAg Levels in HIV/HBV Co-infected Patients with Different HBeAg Statuses

	HBeAg positive			HBeAg negative		
	PM (N=183)	Non-PM (N=93)	*p value*	PM (N=133)	Non-PM (N=25)	*p value*
HBV DNA (Log10,IU/L)	7.91 (7.28,8.7)	8.3 (7.6,8.7)	**0.027**	6.74 (4.96,7.99)	6.48 (4.56,7.75)	0.641
HBsAg (COI)	1998 (1234.5,4105.5)	1222 (509.9,3928)	**<0.001**	6700.5 (2688,7753.75)	4569 (2158,6307)	**0.015**

Data are presented as median (P25 - P75). P values are using the Mann-Whitney U test for continuous variables. The p value of less than 0.05 represents a statistically significant difference. Abbreviations: PM: with Point Mutations; Non-PM: Without Point Mutations; COI: cut off index.

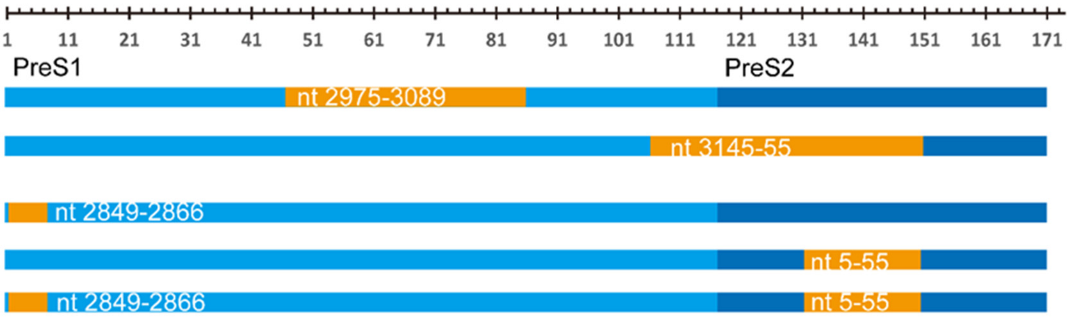

Figure S1. Diagram of Five Common High-Frequency Deletion Mutant Strains. The scale represents amino acid positions. Blue bars indicate the PreS region, with shades distinguishing S1 and S2. Orange bars represent deletion sites, and specific nucleotide positions of deletions are labeled in white.

Table S2. Number of Available Data for Each Indicator at Each Follow-up Time Point

	Baseline	1 year	2 years	3 years	4 years	5 years	6 years
**PM**							
APRI	317	294	276	225	186	164	117
Fib-4	317	294	275	224	186	164	117
AFP	315	62	69	70	94	73	71
ALT	317	304	285	237	195	166	119
AST	317	304	285	237	195	166	119
PLT	317	304	285	237	195	166	119
**Non-PM**							
APRI	118	110	102	86	75	62	47
Fib-4	118	110	102	86	75	62	47
AFP	116	22	29	24	29	25	29

ALT	118	114	106	89	77	64	48
AST	118	114	106	89	77	64	48
PLT	118	114	106	89	77	64	48
**PreS1**							
APRI	46	46	46	46	46	46	46
Fib-4	46	46	46	46	46	46	46
AFP	44	2	4	5	16	12	15
ALT	46	43	41	38	35	33	24
AST	46	43	41	38	35	33	24
PLT	46	43	41	38	35	33	24
**PreS2**							
APRI	22	20	19	13	11	8	7
Fib-4	22	20	19	13	11	8	7
AFP	22	5	7	9	5	4	3
ALT	22	21	19	13	11	9	7
AST	22	21	19	13	11	9	7
PLT	22	21	19	13	11	9	7
**PreS1+2**							
APRI	25	26	26	23	22	18	12
Fib-4	25	26	26	23	22	18	12
AFP	25	3	6	11	12	6	7
ALT	27	27	27	26	22	19	13
AST	27	27	27	26	22	19	13
PLT	27	27	27	26	22	19	13
**w/o del**							
APRI	340	316	294	239	194	168	122
Fib-4	340	316	293	238	194	168	122
AFP	340	74	81	69	90	76	75
ALT	340	327	304	249	204	169	123
AST	340	327	304	249	204	169	123
PLT	340	327	304	249	204	169	123

APRI, aspartate aminotransferase to platelet ratio index; Fib-4, fibrosis-4 index; AFP, alpha-fetoprotein; ALT, alanine aminotransferase; AST, aspartate aminotransferase; PLT, platelet count.

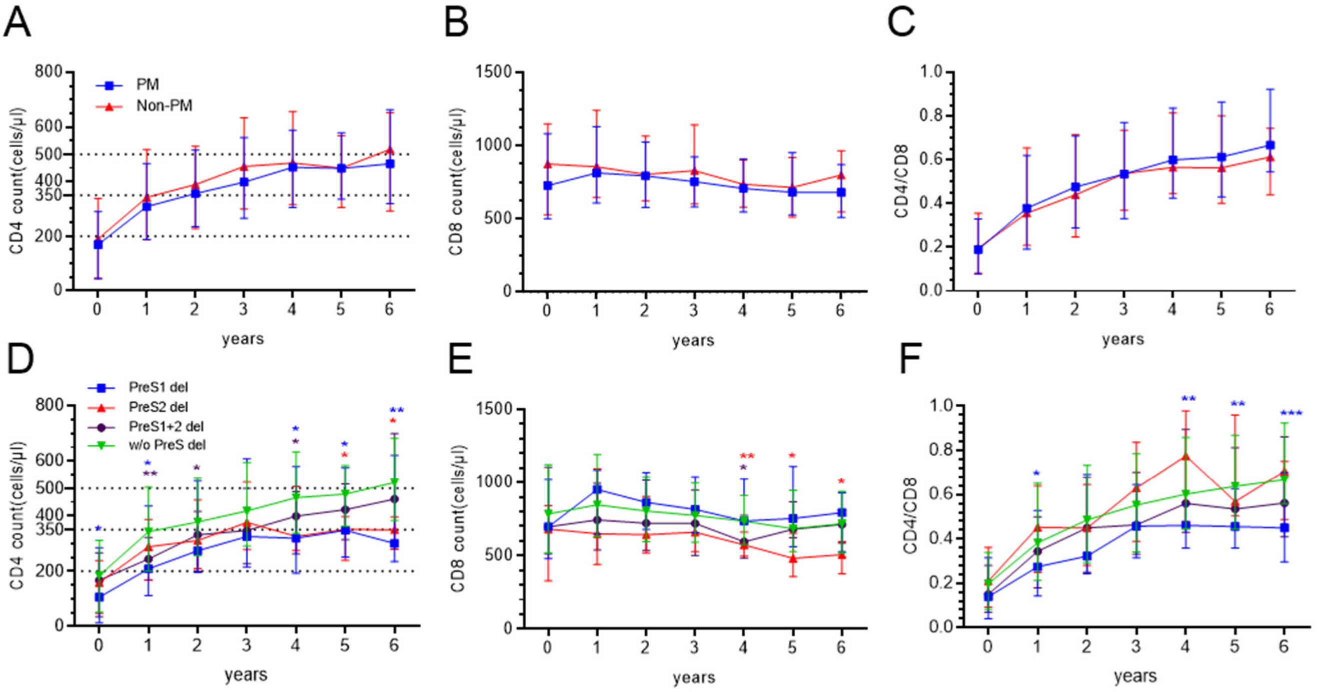

Figure S2. The impact of HBV Point Mutations and PreS deletion mutants on immune reconstitution in People living with HIV and HBV co-infection. (A, B, C) CD4+T cell counts, CD8+T cell counts and CD4/CD8 ratio among PM and Non-PM groups before treatment and 1-6 years of ART treatment. (D, E, F) CD4+T cell counts, CD8+T cell counts and CD4/CD8 ratio among groups before treatment and 1-6 years of ART treatment.Blue asterisks indicate the comparison between "PreS1 del" and "w/o del"; Red asterisks indicate the comparison between "PreS2 del" and "w/o del"; Purple asterisks indicate the comparison be-tween "PreS1+2 del" and "w/o del". The Mann-Whitney U test for comparison between groups.*p < 0.05, **p < 0.01 ,***p < 0.001.
